# Ventricular tachycardia exit site location within myocardial reinnervation zones predicts hemodynamic stability in ischemic cardiomyopathy

**DOI:** 10.1007/s10840-026-02255-4

**Published:** 2026-02-19

**Authors:** Jorge L. Reyes, Darshan Krishnappa, Lucy Q. Lin, Faris Haddadin, David G. Benditt, Venkatakrishna N. Tholakanahalli

**Affiliations:** 1 Present Address: Advanced Interventional Cardiac Electrophysiology, LAA Closure Program, Cardiovascular Division, Minneapolis VA Health Care System, University of Minnesota, Minneapolis, MN 55417 USA; 2 Minneapolis VA Health Care System, Minneapolis, MN USA; 3 Cardiology Division, Oregon Health Sciences University, Portland, OR USA

**Keywords:** Ventricular tachycardia, Ischemic cardiomyopathy, Hemodynamic stability, Autonomic nervous system, Cardiac electrophysiology

## Abstract

**Purpose:**

Hemodynamic stability during ischemic ventricular tachycardia (VT) critically influences mapping strategies and ablation outcomes. This study investigated whether VT exit site location within distinct myocardial scar zones affects hemodynamic stability.

**Methods:**

We analyzed 51 VTs in 27 patients with ischemic scar-mediated VT who underwent electroanatomic mapping and catheter ablation (2014–2017). Exit sites were categorized by voltage mapping as adjacent to central scar zone (CSZ, bipolar voltage < 0.5 mV) or peri-infarct border zone (PIBZ, voltage 0.5–1.5 mV). Hemodynamic stability was defined as sustained VT without precipitous blood pressure drop requiring cardioversion. Multivariate logistic regression identified independent predictors of instability.

**Results:**

Of 51 VTs, 30 (58.8%) were hemodynamically unstable. Among stable VTs, 81% had PIBZ exit sites versus 19% CSZ exits (*P* = 0.01). CSZ exit site location independently predicted hemodynamic instability (OR 6.8, 95% CI 1.3–35.8; *P* = 0.02) after adjusting for cycle length and ejection fraction. Unstable VTs had lower ejection fraction (28.7 ± 10.5% vs. 35.7 ± 11.4%, *P* = 0.03) and shorter cycle length (330.5 ± 78.5 ms vs. 403.2 ± 107.7 ms, *P* = 0.007).

**Conclusions:**

VT exit site location adjacent to distinct scar zones independently predicts hemodynamic stability. CSZ exits are associated with instability, while PIBZ exits maintain stability. These findings may reflect differential post-infarction reinnervation patterns and mechanical forces between scar zones, enhancing risk stratification and guiding ablation strategy selection in ischemic VT.

**Graphical Abstract:**

**Figure:**
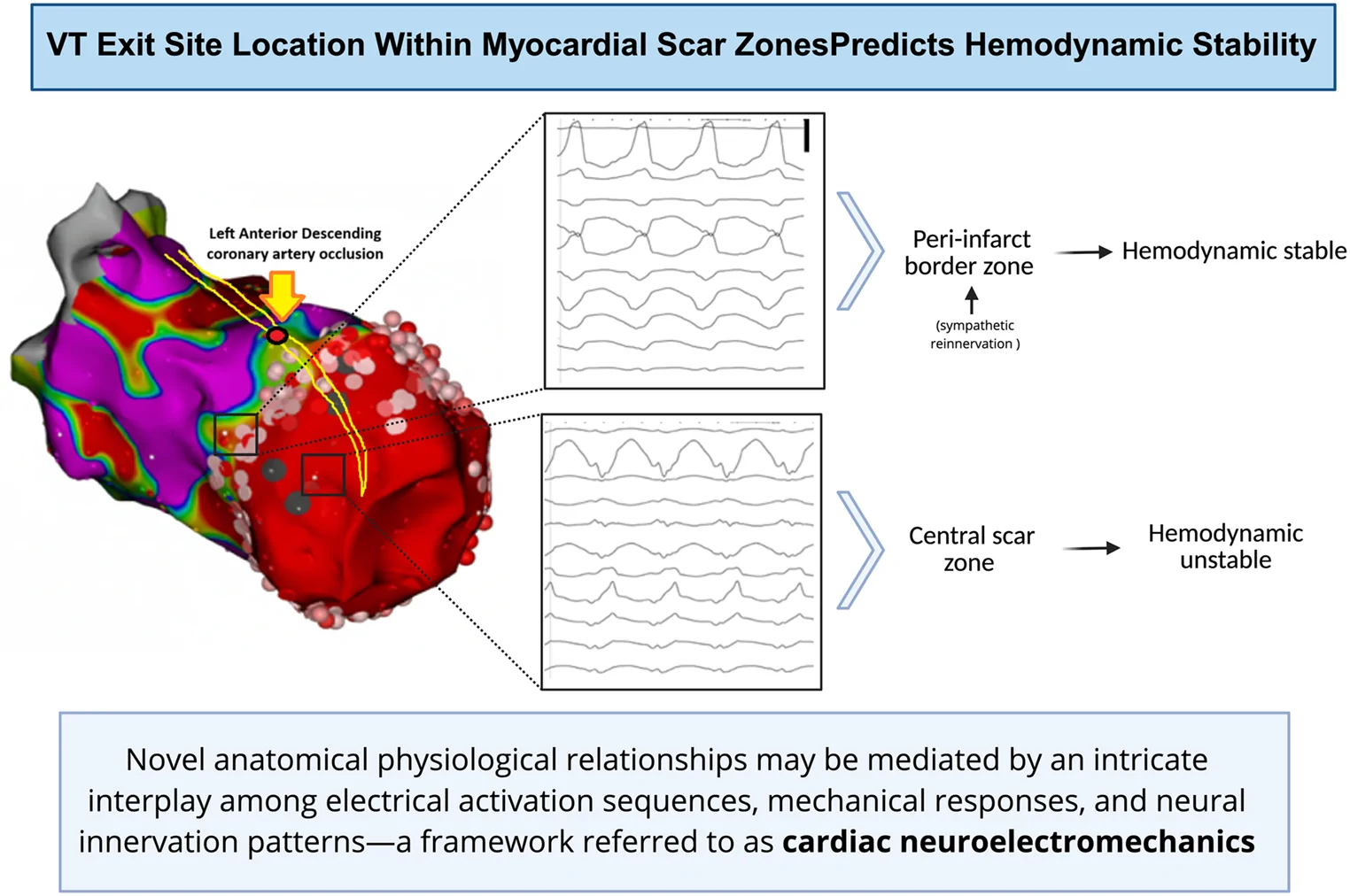

## Introduction

Ischemic scar-mediated ventricular tachycardia (VT), occurring in the chronic phase after myocardial infarction, represents the most common form of scar-related VT and confers substantial morbidity and mortality through increased risk of sudden cardiac death and heart failure exacerbation [1]. While implantable cardioverter-defibrillators (ICDs) improve survival in both primary and secondary prevention settings, ICDs do not prevent arrhythmia recurrence and consequently do not mitigate impact on patients of hemodynamically unstable VT. Catheter ablation has emerged as an important strategy for VT management, with demonstrated reductions in VT recurrence and mortality [2]. 

Arrhythmia stability is a critical determinant of the mapping and ablation approach to ischemic VT. Hemodynamically stable VT allows comprehensive activation and entrainment mapping to identify precisely critical isthmuses and exit sites [3]. In contrast, unstable VT necessitates alternative mapping strategies that are less precise [4, 5]. However, despite the important clinical implications of hemodynamic instability during VT, the determinants of instability remain incompletely understood.

Post-infarction cardiac reinnervation follows a distinct spatial pattern characterized by regional heterogeneity in sympathetic nerve regeneration. The central scar/core infarct zone consists of dense fibrotic tissue with minimal sympathetic reinnervation due to the presence of inhibitory molecules (chondroitin sulfate proteoglycans), lack of viable myocytes, and an environment largely resistant to nerve regrowth [6–8]. In contrast, the peri-infarct (border) zone surrounding the core demonstrates heterogeneous sympathetic reinnervation with areas of both denervation and hyperinnervation, where nerve sprouting occurs due to upregulation of growth factors such as nerve growth factor (NGF) [9, 10]. 

We hypothesized that VT exit site location within or adjacent to these anatomically and functionally distinct post-infarct zones influences hemodynamic stability. Specifically, we postulated that exit sites located adjacent to the central scar zone (CSZ) would tend to be associated with hemodynamic instability as they exhibit minimal post-infarct reinnervation and by being tethered by scar are prone to electrophysiologic effects wall tension derived from nearby non-infarcted myocardium.

On the other hand, exit sites in the peri-infarct border zone with re-innervation (albeit heterogeneous), and being comprised of greater proportion of normal tissue that is less prone to activation by mechanical forces, would be associated with more stable VT. This study investigates this novel neuroanatomical-physiological relationship and its implications for VT ablation.

## Methods

### Study population

We conducted a retrospective analysis of patients with ischemic scar-mediated VT who underwent catheter ablation at our institution between 2014 and 2017. The study protocol was approved by our Institutional Review Board (IRB NUMBER 1594880).

Demographic and clinical data were recorded for each patient, including age, sex, comorbidities, medication usage, left ventricular (LV) function, and dimensions as assessed by pre-procedural echocardiography. The culprit coronary vessel responsible for the index myocardial infarction was identified through electronic health record review and coronary angiography. Details of prior revascularization were documented. Scar burden and LV remodeling were assessed using bipolar (0.5 to 1.5 mV) and unipolar voltage (1.5 to 8.3 mV) mapping [11]. Specifically central core fibrosis exhibited bipolar voltage ≤ 0.5 mV and unipolar voltage ≤ 1.5 mV. Other low voltage regions (0.5 to 1.5 mV) were categorized as peri-infarction border zone (PIBZ).

### Electrophysiology study and VT characteristics

All electrophysiology studies and ablations were performed under general anesthesia with standardized hemodynamic support protocols, including vasopressor and inotropic agents as needed to maintain mean arterial pressures above 60 mmHg. Electroanatomic mapping utilized the CARTO 3D^®^ mapping system (Johnson & Johnson Med Tech, Irvine, CA, USA). A multipolar catheter (Pentaray ^®^, Johnson & Johnson Med Tech, Irvine, CA, USA) was used for high-density mapping to delineate scar substrate. A 3.5 mm irrigated tip catheter (Thermacool Smart-Touch, Johnson & Johnson Med Tech, Irvine, CA, USA) was used for ablation.

For each VT, we documented morphology, cycle length (CL), and hemodynamic stability. Programmed electrical stimulation for VT induction was performed after completion of substrate mapping in all cases, using a standardized protocol with up to triple extrastimuli from the right ventricular apex and outflow tract. Hemodynamic instability was defined as unstable VTs with systolic blood pressure precipitously dropping below 70 mmHg requiring emergency cardioversion.

Only clinical VTs were included in this analysis. Clinical VT was defined as ventricular tachycardia induced during the electrophysiology procedure that matched documented 12-lead ECG morphologies from prior clinical episodes, including ICD electrograms, telemetry recordings, or emergency department presentations. In patients without prior VT documentation, the most frequently induced VT morphology during the electrophysiology study was designated as the clinical VT. Non-clinical VTs induced during programmed stimulation but lacking morphologic correlation with previously documented arrhythmias were excluded.

### Scar zone classification

**Fig. 1:**
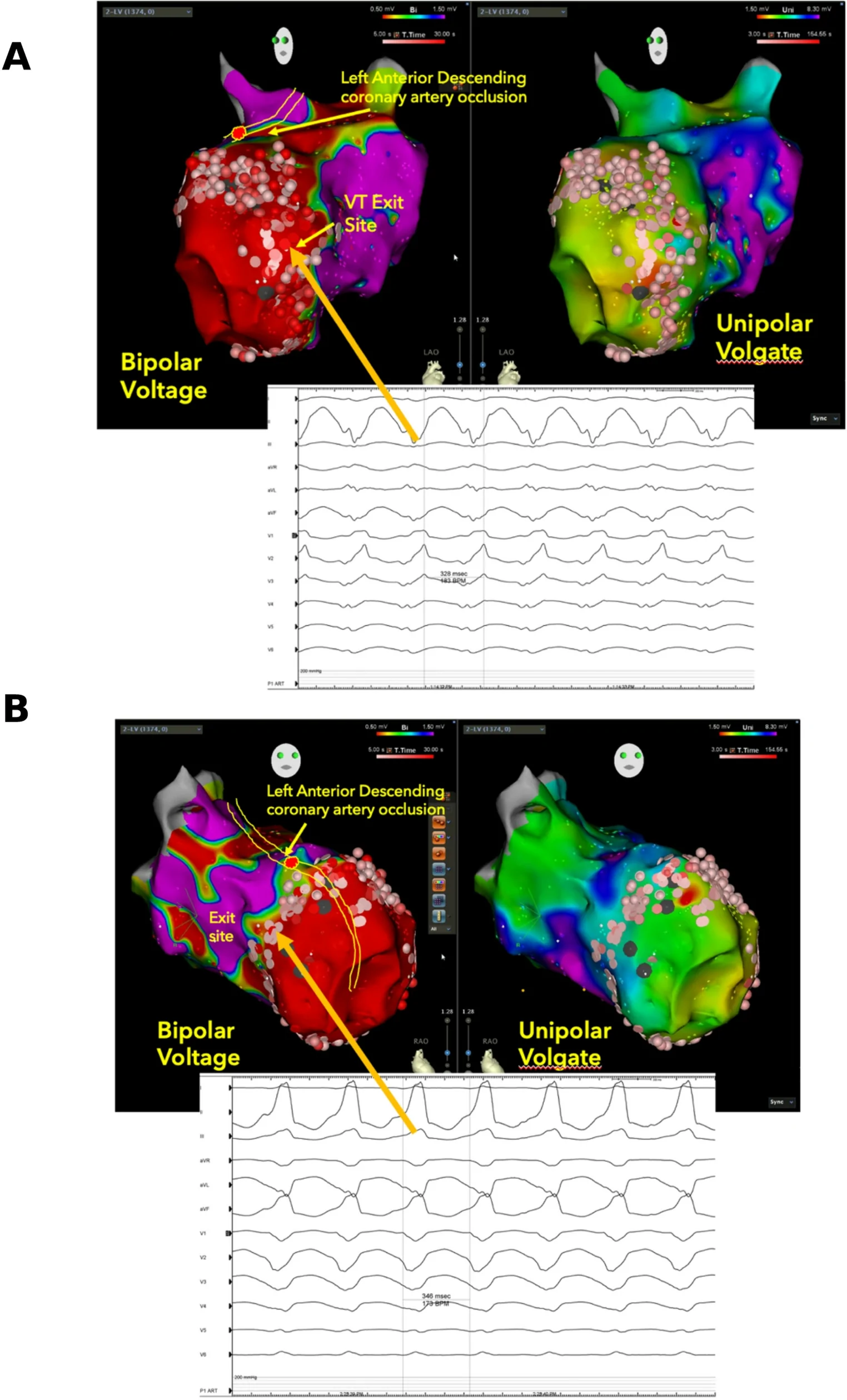
Representative electroanatomic voltage maps demonstrating the relationship between VT exit site location and hemodynamic stability. **Panel A**: Hemodynamically unstable VT with exit site located in the central scar zone (yellow arrow). The exit site is positioned within dense scar tissue (red, < 0.5 mV) in the core of the infarct. **Panel B**: Hemodynamically stable VT with exit site located in the peri-infarct border zone (yellow arrow). The exit site is positioned within intermediate voltage tissue (yellow-green, 0.5–1.5 mV) surrounding the dense scar core. Both panels show bipolar (left) and unipolar (right) voltage maps with corresponding electrograms demonstrating VT morphology. The spatial relationship illustrates that VT exit sites in the peri-infarct border zone are associated with hemodynamic stability, while exit sites in the central scar zone are associated with hemodynamic instability during ventricular tachycardia

To systematically categorize exit sites in relation to post-infarction anatomical zones, we developed a classification scheme based on established voltage mapping criteria, as illustrated in Fig. 1.

Exit site localization methodology varied based on hemodynamic tolerance. For stable VTs, exit sites were defined using activation mapping and entrainment mapping. For unstable VTs requiring prompt termination, exit sites were identified using systematic pace mapping during sinus rhythm, with the exit site defined as the location demonstrating optimal pace map concordance (11/12 leads) with the documented clinical VT morphology, complemented by the presence of late potential signals during sinus rhythm or ventricular pace mapping. The exit site was categorized based on its location within two distinct scar zones: CSZ, dense scar tissue with bipolar voltage < 0.5 mV, representing the fibrotic core of the infarct; PIBZ, intermediate voltage tissue (0.5–1.5 mV) surrounding the central core, representing the heterogeneously scarred border region.

### Statistical analysis

Continuous variables were expressed as mean ± standard deviation, and categorical variables as frequencies and percentages. Continuous variables were compared using Student’s t-test, while categorical variables were compared using chi-square or Fisher’s exact test.

Multivariate logistic regression analysis identified factors predicting hemodynamic instability during VT. Variables with *p* < 0.1 on univariate analysis were included in the multivariate model. Model accuracy was assessed by calculating the area under the receiver-operating characteristic curve. Statistical significance was defined as *p* < 0.05. All analyses were performed using SPSS version 23 (IBM Corporation, Chicago, USA).

## Results

### Patient and VT characteristics

We analyzed 51 distinct VTs in 27 patients with ischemic scar-mediated VT. All patients were male. Overall, 30 VTs (58.8%) were hemodynamically unstable, while 21 (41.2%) were stable. Table 1 presents the baseline clinical characteristics of patients according to VT hemodynamic stability.

**Table 1:** Baseline characteristics of patients undergoing VT ablation

		Hemodynamically unstable VT (*n* = 30)	Hemodynamically stable VT (*n* = 21)	*p*-value
Age (in years)		67.1 ± 9.2	67 ± 8.2	0.98
Hypertension, n(%)		22 (73.3)	13 (61.9)	0.39
Diabetes mellitus, n(%)		12 (40)	15 (71.4)	0.03
Chronic kidney disease, n(%)		15 (50)	5 (23.8)	0.06
Anti-arrhythmic drugs, n (%)	Betablockers	29 (96.7)	20 (95.2)	0.8
	Amiodarone	16 (53.3)	9 (42.9)	0.46
	Mexiletine	17 (56.7)	5 (23.8)	0.02
	Ranolazine	2 (6.7)	1 (4.8)	0.78
Left ventricular ejection fraction (%)		28.7 ± 10.5	35.7 ± 11.4	0.03
Left ventricular end diastolic dimension (in mm)		65.7 ± 11.7	61.3 ± 12.6	0.32

All continuous variables expressed as Mean ± Standard deviation

*VT* Ventricular tachycardia

Patients with hemodynamically unstable VT had significantly lower left ventricular ejection fraction (28.7 ± 10.5% vs. 35.7 ± 11.4%, *p* = 0.03) and more frequent mexiletine use (56.7% vs. 23.8%, *p* = 0.02). VT cycle length was significantly shorter in the hemodynamically unstable group (330.5 ± 78.5 ms vs. 403.2 ± 107.7 ms, *p* = 0.007). The prevalence of diabetes mellitus was significantly higher in patients with hemodynamically stable VT (71.4% vs. 40%, *p* = 0.03) (Table 1).

### Association between exit site scar zone and hemodynamic stability

We found a strong relationship between VT exit site location within scar zones and hemodynamic stability (Table 2). Among hemodynamically stable VTs, 17 (81%) had exit sites in the PIBZ, while only 4 (19%) had exits in the CSZ (*p* = 0.01). Conversely, among hemodynamically unstable VTs, 16 (53.3%) had exit sites in the CSZ, while 14 (46.7%) had exits in the PIBZ.

**Table 2:** Substrate characteristics of patients undergoing VT ablation

		Hemodynamically unstable VT (*n* = 30)	Hemodynamically stable VT (*n* = 21)	*p*-value
Culprit vessel, n (%)	LAD	11 (36.7)	7 (33.3)	0.21
	RCA	17 (56.7)	9 (42.9)	
	LCX	2 (6.7)	5 (23.8)	
Scar burden (%)		21 ± 7.3	23.6 ± 17.8	0.48
Ventricular tachycardia cycle length (in milliseconds)		330.5 ± 78.5	403.2 ± 107.7	0.007
Location of exit site, n (%)	CSZ	16 (53.3)	4 (19.1)	0.01
	PIBZ	14 (46.7)	17 (81)	

Continuous variables expressed as Mean ± Standard deviation

*LAD* Left anterior descending coronary artery

*RCA* Right coronary artery

*Lcx* Left circumflex coronary artery

*CSZ* Central scar zone

*PIBZ* Peri-infarct border zone

Among hemodynamically stable VTs, those with exit sites in the central scar zone had a higher LV ejection fraction compared to those with exit sites in the peri-infarct border zone (42.5 ± 5% vs. 34.1 ± 12%, *p* = 0.05). No such difference was observed among hemodynamically unstable VTs.

### Multivariate analysis

Binomial stepwise logistic regression identified independent predictors of hemodynamic stability during VT (Table 2). The model was statistically significant (χ²(4) = 24.7, *p* < 0.0005), explained 51.7% (Nagelkerke R²) of the variance in hemodynamic stability, and correctly classified 78.5% of cases.

After adjusting for clinical covariates including tachycardia cycle length and ejection fraction, VT exit site location in the CSZ remained a strong independent predictor of hemodynamic instability (OR 6.8, 95% CI 1.3–35.8, *p* = 0.02). Conversely, diabetes mellitus emerged as an independent predictor of hemodynamic stability (OR 0.11, 95% CI 0.02–0.78, *p* = 0.03).

## Discussion

This study introduces a novel concept related to VT mapping and ablation: specifically a potential neuroanatomical-physiological interaction whereby the location of VT exit sites within distinct post-infarction scar zones critically influences hemodynamic stability during tachycardia. To our knowledge, this is the first investigation demonstrating that exit site location within the CSZ versus PIBZ independently predicts hemodynamic response during VT, suggesting an important interplay between neuroanatomical substrate, scar complexity and electrophysiological activation, and autonomic responses in determining arrhythmia outcomes. While the pathophysiology of VT stability cannot be stated with certainty at this time, it likely reflects a complex interplay of several physiological mechanisms related to variations in scar region mechanics and post-infarction regional reinnervation.

### Possible mechanistic framework based on scar complexity and mechanical forces

A primary potential consideration is the fundamental difference in tissue architecture between the CSZ and PIBZ. The CSZ consists predominantly of dense fibrotic tissue with minimal viable myocytes, creating a stiff, non-compliant substrate that is mechanically tethered to surrounding tissue [12, 13]. This rigid anchoring makes CSZ tissue adjacent to non-infarcted contracting myocardium particularly susceptible to stretch-induced electrophysiological effects [14]. As demonstrated by Gornick et al., mechanical stretch of myocardial tissue can induce significant electrophysiological changes, including altered conduction and increased arrhythmogenicity [15]. In contrast, the PIBZ contains a higher proportion of viable myocytes interspersed with fibrotic strands, resulting in greater tissue compliance and most likely a lesser degree of induced mechanical strain during contraction of adjacent healthy myocardium. On the other hand, the complex network of surviving myocardial fibers within the PIBZ creates multiple potential pathways for wavefront propagation. While the latter anatomy might allow for collision of activation wavefronts causing instability, it may conversely permit more local activation pathways resulting in a more stable scenario [16]. One may speculate that VTs exiting from the PIBZ may be more likely to engage these multiple pathways, leading to more organized myocardial activation and more stable mechanical function. Additionally, the heterogeneous nature of the PIBZ may result in smaller regions of synchronized myocardial activation during VT, limiting the mass of dysfunctionally contracting myocardium and thereby preserving hemodynamic stability [17]. These anatomical and mechanical considerations provide a substrate-based explanation for our seemingly non-intuitive observation that VTs with exit sites in the PIBZ are more likely to maintain hemodynamic stability compared to those exiting from the CSZ.

### Scar zone reinnervation biology

Beyond structural differences, differences in reinnervation patterns following myocardial infarction may possibly help to explain the relationship between exit site location and hemodynamic stability. Myocardial infarction is typically associated with damage to regional nerve endings, particularly along the endocardial surface of the infarct zone [18]. During the subsequent healing process, sympathetic reinnervation occurs in a spatially heterogeneous pattern [19, 20]. The central scar zone develops minimal sympathetic reinnervation due to the presence of inhibitory molecules such as chondroitin sulfate proteoglycans and Growth Associated Protein 43 (GAP43), dense collagen matrix that physically impedes nerve growth, and lack of viable myocytes that normally provide trophic support for nerve sprouting [21, 22]. Conversely, the peri-infarct border zone demonstrates heterogeneous sympathetic reinnervation with areas of both hyperinnervation and denervation. This occurs through upregulation of growth factors like nerve growth factor, presence of partially viable myocardial tissue, and a more permissive microenvironment for nerve regeneration [23, 24]. 

### Proposed neuroelectromechanical model

We propose an integrated model of cardiac neuroelectromechanics that combines both mechanical and neuroanatomical factors. When VT exits from the PIBZ, the initial electrical activation occurs in regions with complex viable tissue networks and often enhanced sympathetic innervation as a consequence of post-MI healing. The subsequent mechanical contraction follows more physiological patterns and activates intact or hyperinnervated sympathetic afferents, triggering a robust cardiac sympathetic reflex that maintains blood pressure and hemodynamic stability. Conversely, when VT exits from the CSZ, the initial activation occurs in regions with minimal viable tissue, disrupted contraction patterns, and minimal sympathetic reinnervation. The mechanical activation of these poorly innervated dense fibrotic areas results in dysfunctional contraction patterns, increased wall stress on near-by adjacent myocardium, and inadequate sympathetic reflex activation, leading to insufficient autonomic compensation and hemodynamic instability.

This integrated framework aligns with previous research by Huikuri et al., who found shorter sinus cycle lengths (indicating greater sympathetic activation) during hemodynamically stable VT compared to unstable VT [25]. This observation supports the concept that robust sympathetic responses protect against hemodynamic compromise during VT [26].

### Diabetes and autonomic remodeling

The independent association between diabetes mellitus and hemodynamic stability during VT in our study provides additional support for the potential role of autonomic neural influences. Diabetic autonomic neuropathy follows a length-dependent pattern, with parasympathetic dysfunction typically preceding sympathetic impairment [27]. Consequently there may be a sympatho-parasympathetic imbalance that may augment sympathetic responses to VT through reduced parasympathetic antagonism, potentially enhancing hemodynamic compensation even in patients with exit sites in less favorable reinnervation zones. This finding is particularly interesting as it suggests that pre-existing autonomic neuropathy may paradoxically be protective against hemodynamic collapse during VT by altering the balance of autonomic inputs to the cardiovascular system during arrhythmia.

### Clinical implications

Our findings have several important clinical implications. Risk Stratification: VT exit site location within scar zones could help identify patients at higher risk for hemodynamically unstable VT later in the mapping/ablation study. Patients with substrate predominantly in central scar zones may benefit from more aggressive ICD programming and earlier consideration of prophylactic ablation. Recognizing exit site location allows operators to anticipate hemodynamic tolerance and prepare appropriate ablation strategies. CSZ exit sites predict instability and favor substrate-based approaches targeting late potentials, channels, and isthmuses. Conversely, PIBZ exit sites suggest greater feasibility of activation and entrainment mapping, justifying investment in these time-intensive techniques. Understanding the scar zone location of potential exit sites guides anesthesia management and hemodynamic support strategies. Exit sites in central scar zones should prompt enhanced hemodynamic monitoring and support, with consideration of mechanical circulatory support availability.

In summary, our findings favor substrate-based ablation approaches in patients with exit sites predominantly in central scar zones, where activation mapping may be limited by hemodynamic instability. Conversely, patients with exit sites in peri-infarct border zones may be better candidates for detailed activation mapping approaches. The importance of scar zone reinnervation patterns suggests potential benefits from cardiac neuromodulation approaches, particularly in patients with recurrent unstable VT originating from poorly reinnervated central scar regions.

### Limitations

Several limitations should be acknowledged. This was a retrospective single-center study with a modest sample size (51 VTs in 27 patients), though our findings achieved statistical significance and demonstrated consistent patterns across multiple variables. Validation in larger prospective cohorts remains essential. Our study population consisted exclusively of males, reflecting the demographic of our veteran population, though this limits generalizability to female patients where sex differences in autonomic function and post-infarction remodeling may influence outcomes. We did not directly assess sympathetic innervation through ¹²³I-MIBG imaging, though our voltage-based scar zone classification aligns with established post-infarction reinnervation biology and has been validated in prior neuroanatomical studies. Detailed autonomic function testing was not performed. However, our clinical endpoint of hemodynamic stability represents the clinically relevant manifestation of autonomic dysfunction during VT. Additionally, antiarrhythmic drugs influence VT cycle length and autonomic responses and were not systematically assessed for their potential confounding effects on our primary endpoint. Our definition of hemodynamic instability, while not standardized, reflects real-world clinical decision-making and has validity for practicing electrophysiologists. Several confounding variables may have influenced our results, including the significantly lower LVEF in the hemodynamically unstable group, though multivariate analysis controlled for ejection fraction and other relevant clinical variables. The retrospective design precluded standardized protocols yet allowed analysis of real-world clinical outcomes in consecutive patients undergoing VT ablation.

## Conclusion

In patients with ischemic scar-mediated VT, exit site location adjacent to distinct myocardial scar zones independently predicts hemodynamic stability. VTs with exit sites adjacent to the CSZ are significantly more likely to cause hemodynamic compromise, while PIBZ exits tend to maintain stability. This novel anatomical-physiological relationship may be mediated by an intricate interplay between electrical activation sequences, mechanical responses and neural innervation patterns —a concept reasonably referred to as cardiac neuroelectromechanics.

## Data Availability

No datasets were generated or analysed during the current study.

## Abbreviations

CSZ: central scar zone
LV: left ventricular/left ventricle
LVEF: left ventricular ejection fraction
MI: myocardial infarction
PIBZ: peri-infarct border zone
VT: ventricular tachycardia
